# Using *Fomitopsis pinicola* for bioinspired synthesis of titanium dioxide and silver nanoparticles, targeting biomedical applications†

**DOI:** 10.1039/d0ra02637a

**Published:** 2020-08-28

**Authors:** Suriya Rehman, Rabindran Jermy, Sarah Mousa Asiri, Manzoor A. Shah, Romana Farooq, Vijaya Ravinayagam, Mohammad Azam Ansari, Zainab Alsalem, Reem Al Jindan, Zafar Reshi, Firdos Alam Khan

**Affiliations:** Department of Epidemic Disease Research, Institute for Research & Medical Consultations, (IRMC), Imam Abdulrahman Bin Faisal University Dammam 31441 Saudi Arabia surrehman@iau.edu.sa suriyamir@gmail.com https://scholar.google.com/scholar?hl=en&as_sdt=0%2C5&q=suriya+rehman; Department of Nano-Medicine Research, Institute for Research & Medical Consultations, (IRMC), Imam Abdulrahman Bin Faisal University Dammam 31441 Saudi Arabia rjermy@iau.edu.sa; Department of Botany, University of Kashmir Srinagar J&K India; Department of Biophysics, Institute for Research & Medical Consultations, (IRMC), Imam Abdulrahman Bin Faisal University Dammam 31441 Saudi Arabia; Deanship of Scientific Research, Department of Nano-Medicine Research, Institute for Research & Medical Consultations (IRMC), Imam Abdulrahman Bin Faisal University P. O. Box 1982 31441 Dammam Saudi Arabia; Department of Microbiology, College of Medicine, Imam Abdulrahman Bin Faisal University Dammam 31441 Saudi Arabia; Department of Stem Cell Research, Institute for Research & Medical Consultations, (IRMC), Imam Abdulrahman Bin Faisal University Dammam 31441 Saudi Arabia

## Abstract

The current study proposes a bio-directed approach for the formation of titanium oxide and silver nanoparticles (TiO_2_ and Ag NPs), using a wild mushroom, *Fomitopsis pinicola*, identified by 18S ribosomal RNA gene sequencing (gene accession no. MK635350) and phenotypic examination. NP synthesis was confirmed by X-ray diffraction (XRD), Fourier transform infrared spectroscopy (FT-IR), diffuse reflectance UV-visible spectroscopy (DR-UV), and scanning and transmission electron microscopy (SEM/TEM). Furthermore, the impact of NPs on *Escherichia coli* and *Staphylococcus aureus* and a human colon cancer cell line (HCT) were evaluated by MIC/MBC and MTT assays, respectively, along with structural morphogenesis by different microscopy methods. The results obtained showed that TiO_2_ and Ag NPs were found to be significantly active, however, slightly enhanced antibacterial and anticancer action was seen with Ag NPs (10–30 nm). Such NPs can be utilized to control and treat infectious diseases and colon cancer and therefore have potential in a range of biomedical applications.

## Abstract 2

This study proposes a bio-directed approach for the formation of titanium oxide and silver nanoparticles (TiO_2_ and Ag NPs), using a wild mushroom, *Fomitopsis pinicola*, identified by 18S ribosomal RNA gene sequencing (gene accession no. MK635350) and phenotypic examination.

## Graphical abstract

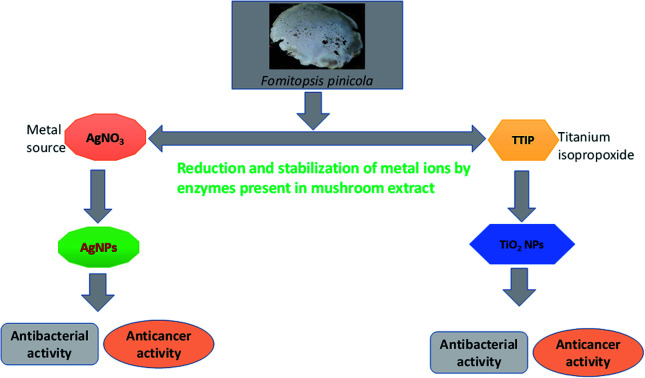

## 1. Background

In recent times, nanotechnology using metal NPs has been an extensively studied field, and involves the transition of bulk material to nanoscale size, introducing a remarkable effect on the properties, from increased surface synergy to quantum restrictions.^1^ Metal NPs like silver, gold, zinc, titanium *etc.* are broadly used in different fields, like agriculture, medicine, biomedical, electronic and other industries.^1–3^ The widely used method for the synthesis of these NPs is mostly chemical, which involves the use of a number of chemicals that could be detrimental to the ecosystem and human health. In contrast, the green synthesis of NPs, using organic sources, like plants, bacteria and fungi, is evolving as an influential field of nanotechnology, due to their safe, eco-friendly and cost-effective properties.^4^ Although, the demand for the green synthesis of NPs has been growing, inadequacy in the information and sources of green methods is preventing competitive chemical synthesis.

Fungi are one of the biological sources, broadly known for bioremediation utility, owing to their capability of mineralizing, a vast array of harmful compounds, with the help of enzymes, secreted to the environment.^5^ Additionally, lower and higher fungi are reported as mediators for the formation of NPs, because they produce huge amount of biomass and are easy to handle.^6–9^ Macrofungi (mushrooms) are excessively loaded in proteins and have been known for their nutrient-rich content and immense medicinal value. Though, only few studies have evaluated the possible application of mushrooms to produce metallic NPs, for which the possible reason could be their seasonal existence, as well as the extreme place of growth. Polyporoids are widely distributed in different biotopes and have been studied as reducing agent for the synthesis of various NPs. During the extracellular synthesis of many NPs like Au, Ag, *Volvariella volvacea*, *Trametes* Fr., *Trametes versicolor*, *Ganoderma lucidum* and *Ganoderma applanatum*, *Pleurotus florida* have been reported as reducing and capping agent.^8,10–12^ Park and Park isolated laccase enzyme from *F. pinicola*, a brown-rot fungus fills Basidiomycota, Agaricomycetes, Fomitopsidaceae, which is the characteristic indicating its eligibility for potential in biological synthesis of NPs.^13^

To the best of the knowledge of authors, the ability of *F. pinicola*, to synthesize metallic NPs has not yet been studied. In the present work, we examined the ability of the wild mushroom sp., *F. pinicola*, to synthesize TiO_2_ and Ag NPs, without using any supplementary reducing agent. The antimicrobial and anticancer potential of synthesized NPs was determined against human pathogens, *E*. coli, *S. aureus* and HCT cell line, respectively.

## 2. Methods

### 2.1. Collection, phenotypic and genotypic studies of *F. pinicola*

For the collection of sporocarps, standard method was followed^14^ (details of sample collection are provided in ESI†). Photographs were taken by Nikon D5300 DSLR Camera with a zoom lens of 18–140 VR. Passport data and the micro-habitat characteristics of collected sample was written in the field book. Sample was properly labeled, given a voucher number and carried to a laboratory for detailed morphometric examination.

Collected specimens were identified by keen observation of structures like pileus, stipe, their shape, structure, gill attachment *etc.*, using standard keys (*e.g.*, Mycokey, Index fungorum *etc.*) field guides and manuals. The samples were dried and deposited at the herbarium of the Centre for Biodiversity and Taxonomy, University of Kashmir, J&K, India. Microscopic features and measurements were made from slides prepared and stained with lactophenol cotton blue, 2% KOH and Melzer's reagent. For spore examination, the spores were tapped off the razor blade onto a clean and a drop of KOH or Melzer's reagent was added. Observation and photographs were captured at magnification between 40× to 100×, using a Nikon Eclipse 80i microscope and phase contrast illumination (Nikon, Tokyo).

### 2.2. DNA isolation and PCR

DNA extraction was done using manual CTAB method (cetyl trimethylammonium bromide).^15^ The extracted DNA was dissolved and preserved in TE (Tris–EDTA) buffer. The DNA was amplified for internal transcribed spacer (ITS) regions using the ITS1 and ITS4 in a PCR System Thermocycler Applied Biosystems with following parameters; denaturation for 10 min at 95 °C, 35 cycles at 95 °C for 1 min, 54 °C for 30 s and 72 °C for 2 min, followed by extension at 72 °C for 10 min. The purification of amplified products was done and sequenced with the same primers.^15^

### 2.3. Sequence and phylogeny analysis

The small subunit sequences were aligned with additional sequences downloaded from NCBI GenBank (http//ncbi.nim.nih.gov) using BioEdit Sequence Alignment Editor (version 7.2.5).^16^ The sequence alignments and phylogenetic analysis were performed using MEGA 10 software (Tamura *et al.* 2011). Phylogeny was studied on ITS-18S rRNA genes in maximum likelihood method. Initial alignment was done using Clustal W software for maximum alignment and minimum gaps. The tree was generated by using the program DNADIST and NEIGHBOR from PHYLIP 3.69.^17^

### 2.4. Biosynthesis of TiO_2_ and AgNPs using *F. pinicola*

**Scheme 1:**
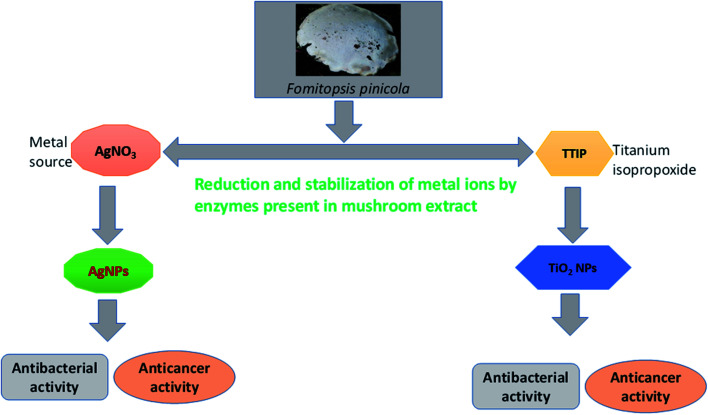
Biosynthesis of TiO_2_ and Ag NPs using the extract of *F. pinicola*.

The synthesis of TiO_2_ and Ag NPs was conducted using the extract of *F. pinicola by* adopting a green synthesis method.^11^ The samples were dried to obtain powder (10 g), which was further mixed with 100 mL of Millipore water and sonicated for 25–30 min. The sonicated mixture was centrifuged at the rpm of 4000 to obtain the clarified solution. Subsequently, the solution was filtered and stored at 4 °C. 10 mL of filtrate was mixed with 1 mM AgNO_3_ (100 mL) and put at room temperature on a shaker for agitation under observation, until the appearance of color change.^11^ A similar procedure was followed for TiO_2_ NPs, where 100 mL of 1 mM of titanium(iv) isopropoxide was used as source solution (Scheme 1). The mushroom extract to 1 mM AgNO_3_ or Ti(OC_3_H_7_)_4_ ratio used for the study was 1 : 10. Finally, the obtained NPs were filtered using Whatman filter paper, washed two times in ethanolic solution and centrifuged (4000 rpm) at 10 °C for 10 min. After drying, the sample was used for further studies.

### 2.5. Characterization of biosynthesized TiO_2_ and AgNPs

The crystalline phase of TiO_2_ and AgNPs was measured using benchtop X-ray diffractometer MiniFlex 600 (Rigaku, Japan). The coordination environment of TiO_2_ and Ag NPs were analyzed using diffuse reflectance UV-visible spectroscopy (V-750, JASCO). The TiO_2_ and AgNPs functional groups were analyzed using Fourier transform infrared spectroscopy (PerkinElmer). The surface morphology, distribution and features of TiO_2_ and AgNPs were studied using SEM (Inspect S50) and TEM (Morgagni 268). For TEM analysis, samples were prepared by dispersing in ethanol followed by shaking in an ultrasonicator for 20 min, and then a suspended drop was dried at room temperature on the carbon-coated copper grid.^18^

### 2.6. Antibacterial activity of biosynthesized NPs

Antibacterial activity of synthesized, TiO_2_ NPs and Ag NPs were studied against the human pathogenic Gram-negative bacteria and Gram positive bacteria, (*E. coli* ATCC35218 and *S. aureus* ATCC29213, respectively) by broth dilution method. The bacterial strains were maintained and nutrient agar media (NA). In preparation for the study, a homogeneous suspension of NPs was prepared by sonication for 15–20 min at 30 °C, ranging in the concentration from 250 to 15.62 μg mL^−1^. Mueller–Hinton (MHB) was used to grow test organisms for overnight at 37 °C and subsequently adjusted to the cell frequency of 10^6^ CFU mL^−1^. The adjusted inoculum of each bacterial strain was added to the solution of MHB with NPs and incubated with shaking at 37 °C for 24 h. Untreated bacteria was used as a negative control. The MIC was recorded as the least concentration of NPs, which had no growth visible in the broth (absence of turbidity).

Following the MIC evaluation, MBC was obtained by taking an aliquot of the MIC for further plating on the MHA plates. The inoculated plates were further incubated for overnight at 37 °C and the MBC was taken as the concentration at which no growth or CFU less than 3 was obtained.^19^

### 2.7. Study of topological changes in treated bacteria

Additionally, the treated culture of *E. coli* and *S. aureus* were studied by SEM for the morphological and physiological alteration caused by NPs. Precisely, adjusted bacterial cells were treated at the concentration obtained as its MIC and subjected to incubation at 37 °C for overnight. Untreated samples were included as the negative control. Later treated and untreated cells were centrifuged at 12 000 rpm for 10 min. The harvested cells were thrice washed using PBS and primarily fixed using 2.5% glutaraldehyde for 4 h, then again fixed with 1% osmium tetroxide for 1–2 h. Cells were washed multiple times and further dehydrated by varying conc. of ethanol (50%, 70%, 90%, 100%). The cells were placed on aluminum stubs and dried using desecrator. Finally, gold coating was done and cells were examined by SEM at an accelerating voltage of 20 kV.^19^

### 2.8. Antiproliferative activity

#### 2.8.1. Cell culture & treatments

Human colorectal carcinoma cells (HCT-116) were used for the study. DMEM medium was used which was supplemented with 10% fetal bovine serum (FBS); (10%) l-glutamine; 10% selenium chloride; 120 μg mL^−1^ and streptomycin; and 120 units per mL penicillin in 5% CO_2_ incubator (Thermo Scientific Heracell-150) at temperature 37°Celsius. The cells with more than 70–80% confluence was used for the TiO_2_ and AgNPs treatments. The treatment of HCT-116 cells were carried out with different concentrations of 0.5 to 8.0 μg mL^−1^. The cells were observed after the time span of 48 h. Experiment was carried out in triplicate for statistical analysis.^20^

#### 2.8.2. Cancer cell morphology

The cell morphology of untreated and treated HCT-116 cells were examined post 48 h under inverted microscope (TS100F-Eclipse, Nikon) and compared the under 200× magnifications.

#### 2.8.3. Cytotoxicity by MTT assay

The cells with confluency of 70–80% in 96-well cell culture plates were subjected to MTT assay. After 48 h, MTT (5 mg mL^−1^) was added in all the wells and kept for 4 h. Later, DMSO was added and plate was read in ELISA Plate Reader 570 nm wavelength (Biotek Instruments, USA).^20^ The (%) percentage of cell viability was calculated as per given formula:
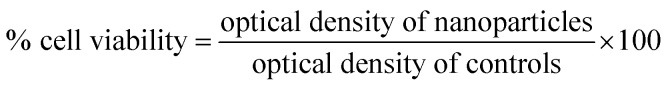


#### 2.8.4. Nuclear staining by DAPI

The cells were stained with DAPI staining to study the effect of TiO_2_ NPs and Ag NPs on the cell nucleus. After 48 h, the treated and untreated HCT-116 cells were immersed in an ice-cold (4%) paraformaldehyde. Later, the cells were added with Triton X-100 prepared in PBS for 5 min to premetallize the cell membrane. The cells were stained using DAPI (5 μg mL^−1^) in PBS, prepared in dark. Washing with Triton X-100 was done, followed by examining the nuclear morphology under confocal scanning microscope (Zeiss, Germany) equipped with digital camera.^20^

In the present study, cell viability data were presented as mean (±) standard deviation (SD) which were obtained from three independent experimental repeats. One-way ANOVA followed by Dunnett's *post hoc* test with GraphPad Prism software (GraphPad Software, Inc., La Jolla, CA, USA) for the statistical analysis. *P* < 0.05 was considered to indicate a statistically significant difference.

## 3. Results

### 3.1. Phenotypic and genotypic studies of *F. pinicola*

**Fig. 1:**
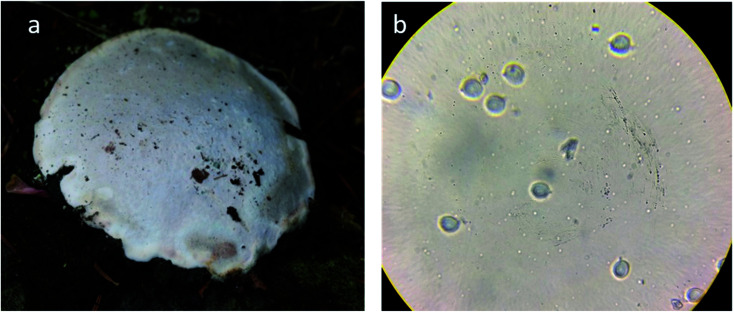
Photographs of brown rot basidiomycetes: *F. pinicola*, (a) under view showing pores (b) basidiospores at 40×.

**Fig. 2:**
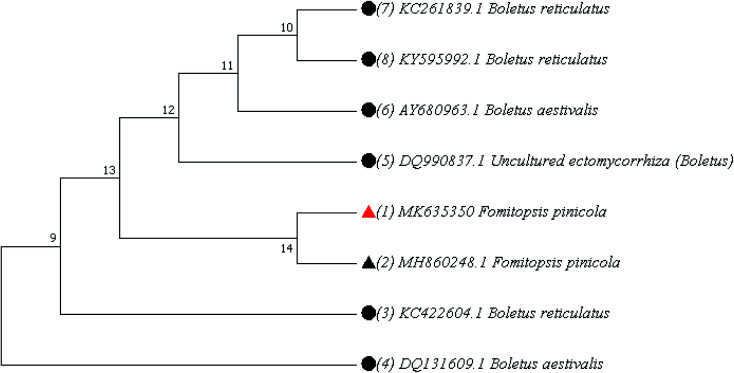
Phylogenetic tree of *F. pinicola* indicated as 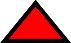.

The phenotypic analysis of mushroom namely *F. pinicola* was done. Spores are 5.5–7 × 4.0–5.0 μm, oval, smooth: spore print pale yellow. Spores are bilaterally symmetrical. The shape of the hilar appendix is beaked (Fig. 1a and b). This was further confirmed by phylogenetic analysis of the ITS1–ITS4 sequences of the mushroom, which was deposited in the NCBI Gene Bank under accession number MK635350. The phylogenetic relationships with related species have been shown in Fig. 2, presenting the similarity score of 100% to *F. pinicola* (MH860248.1) (Table 1).

**Table 1:** Gene bank accession numbers and top BLAST match sequences of the mushroom isolates along with maximum identity, query coverage

Accession number	BLAST match sequence		
	Reference accession number	Coverage	Maximum identity
MK635350	MH860248.1 *Fomitopsis pinicola*	100%	100%
	KC422604.1 *Boletus reticulatus*	99%	98.75%
	DQ131609.1 *Boletus aestivalis*	99%	98.75%
	DQ990837.1 Uncultured mycorrhiza	98%	98.99%
	KY595992.1 *Boletus reticulatus*	91%	99.18%
	AY680963.1 *Boletus aestivalis*	89%	98.88%
	KC261839.1 *Boletus reticulatus*	71%	99.47%

### 3.2. Characterization of biosynthesized TiO_2_ and AgNPs

**Fig. 3:**
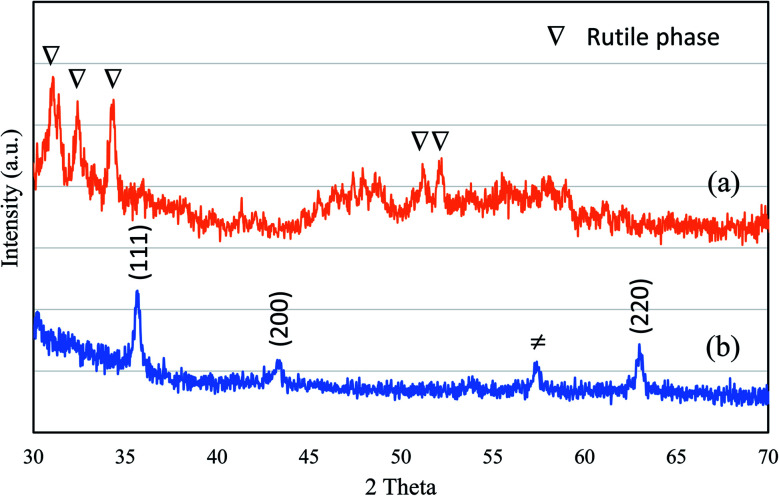
X-ray diffraction spectra of (a) TiO_2_ and (b) Ag NPs.

The production of TiO_2_ and Ag NPs mediated by *F. pinicola* was indicated by examining the color change in the reaction mixture. Fig. 3 shows the XRD spectra of (a) TiO_2_ and (b) AgNPs. The formation of crystalline TiO_2_ with sharp peaks corresponding to rutile phase was observed using the extract of *F. fomentarius*. In case of TiO_2_ synthesis using *F. pinicola* (Fig. 3a), a reduced crystallinity and broadness of peaks indicates nanosized TiO_2_ formation with major phase of rutile. Fig. 3b shows the XRD spectra of Ag NPs. The spectra showed a clear diffraction lines corresponding to (111), (200) and (220) planes indicating the presence of Ag NPs.^21^ The presence of unidentified peak at about 57.5° could be attributed to the crystalline components present in the extract.

**Fig. 4:**
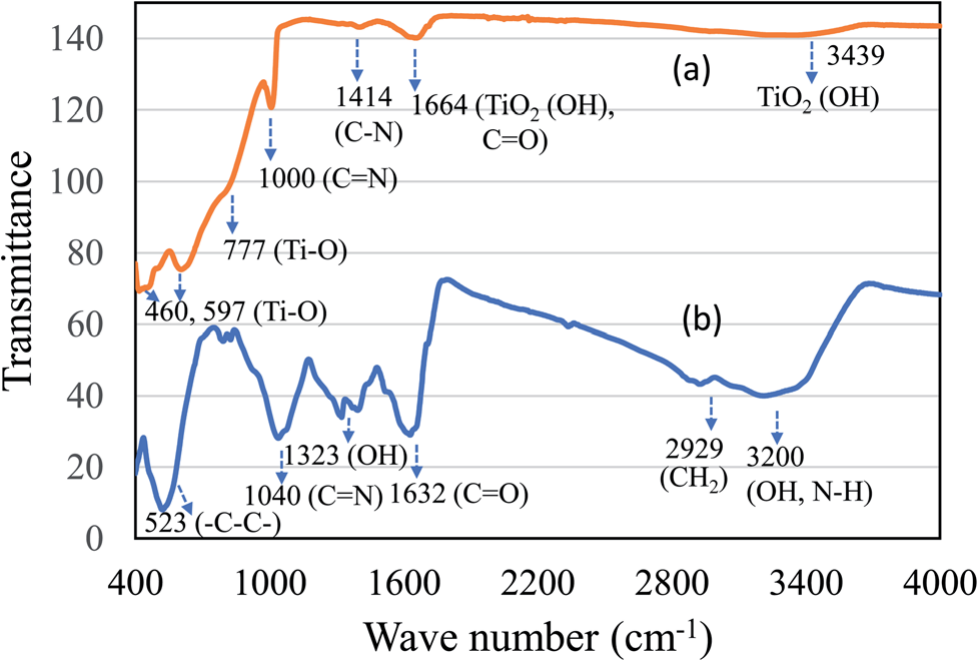
FT-IR spectra of (a) TiO_2_ and (b) Ag NPs.

The presence of active ingredients like polyphenols and flavonoids in the mushroom extract play a key role as bio reductant in reducing metal ions of metal source to nanoparticles. Fig. 4 shows the FT-IR spectroscopy of (a) TiO_2_ and (b) Ag NPs. In case of TiO_2_, the hydroxyl functional group absorption coordinated TiO_2_ species was observed with distinct peaks at 1664 cm^−1^ and 3480 cm^−1^. The presence of TiO_2_ was further confirmed with absorption peak of Ti–O bands at about 460, 597 and 777 cm^−1^. In case of Ag NPs, presence of difference functional groups corresponding to N–H, O–H, and methylene C–H was observed between 1000–3700 cm^−1^ (Fig. 4b). A distinct broad peak appears between 3650–2400 cm^−1^ corresponding to hydroxyl (–OH), N–H stretching of primary amines and methylene (CH_2_). The presence of asymmetrical C–O stretching peak was observed 1632 cm^−1^. The presence of aromatic ring (–C–C–) and aliphatic amine (C–N) was clearly seen with an intense absorption peak at about 523 cm^−1^ and 1040 cm^−1^. In both cases, the presence of elongated peaks at fingerprint region between 1000–1800 cm^−1^ showed several functional group moieties of mushroom extract. The linear aliphatic amines (C–N) showed peak absorption at 1000–1040 cm^−1^. The functional group analysis study using FT-IR indicates that active components (amino, hydroxyl and methyl) in mushroom act as an effective bio reductant to produce TiO_2_ and Ag NPs.

**Fig. 5:**
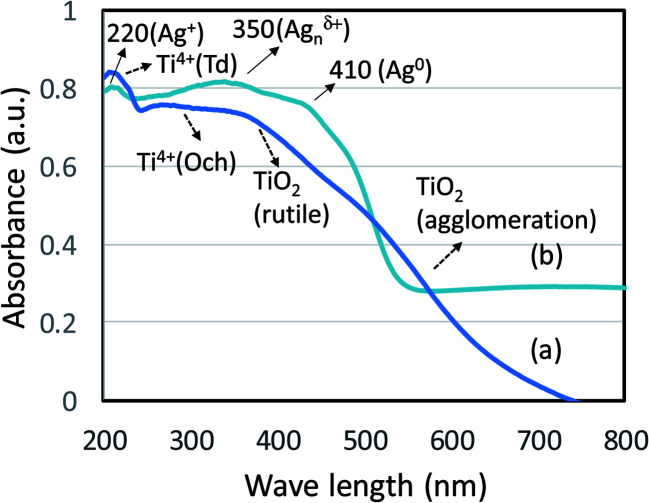
(a and b) shows the diffuse reflectance spectra of TiO_2_ and Ag NPs.

The coordination sites of TiO_2_ and Ag NPs prepared using *F. pinicola* was studied using diffuse reflectance UV-visible spectroscopy. In case of TiO_2_ NPs, isolated tetrahedral Ti^4+^ species was observed with a band at about 220 nm (Fig. 5a). The presence of distinct peaks corresponding to rutile and octahedral Ti compounds were observed between 250–420 nm. The presence of anatase (titania) phase was also reported at about 350 nm. The result coincides with XRD analysis, which indicated the presence of rutile and anatase phase of TiO_2_. The presence of few agglomerated TiO_2_ species were also observed between 500–600 nm. In case of Ag NPs, the sample exhibited presence of three kinds of Ag species indicating the variation in coordination environments (Fig. 5b). A weak absorption at about 224 nm indicates presence of few Ag^+^ species. However, the strong absorption at 352 nm showed presence of Ag_*n*_^*δ*+^ nanoclusters as predominant species followed by Ag^0^ with absorption maxima ranging between 410–540 nm.

**Fig. 6:**
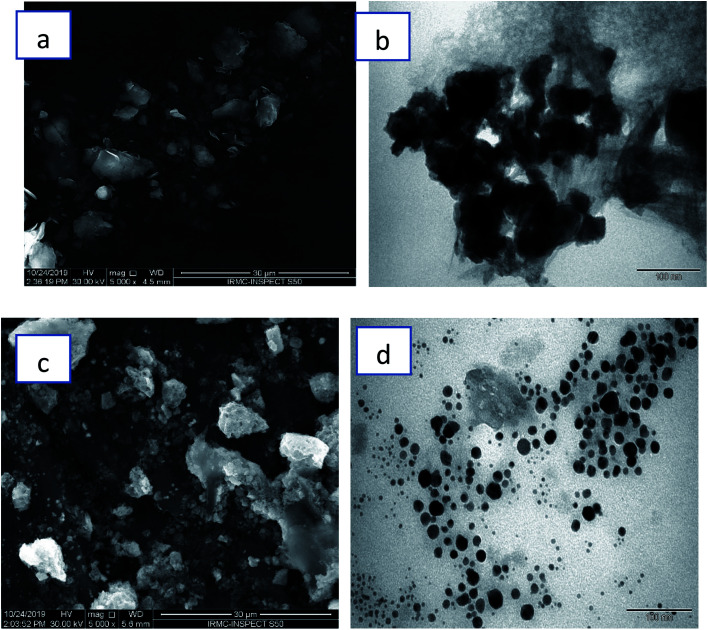
(a) SEM and (b) TEM images of TiO_2_ NPs; (c) SEM and (d) TEM images of Ag NPs.

Fig. 6 demonstrated SEM and TEM morphology of TiO_2_ and Ag NPs. For TiO_2_ NPs, surface features and distribution have been examined through SEM (Fig. 6a). SEM micrographs showed that these nanoparticles are dispersed in irregular distribution with rough surface. TEM (Fig. 6b) showed irregular shape and size. On the other hand, Fig. 6c illustrated spherical Ag nanoparticles with some agglomeration of nanoparticles, while TEM (Fig. 6d) depicted well distribution of small spherical nanoparticles with average diameter ranging from 10–30 nm.

### 3.3. Antibacterial activity of synthesized NPs

**Fig. 7:**
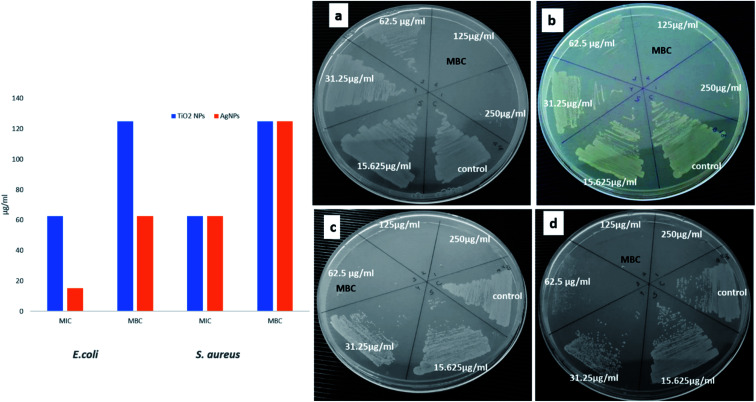
MIC/MBC assay: (a) *E. coli* and (b) *S. aureus* treated with different conc. of TiO_2_ NPs: (c) *E. coli* and (d) *S. aureus* treated with different conc. of Ag NPs.

The assay was performed by examining the MIC and MBC values, against *E. coli and S. aureus.* On treatment with TiO_2_ NPs, the MIC/MBC values were 62.5/125 and 62.5/125 μg mL^−1^ for *E. coli* and *S. aureus*, respectively (Fig. 7a and b). Whereas, Ag NPs, the MIC/MBC values obtained were 15.62/62.5 and 62.5/125 μg mL^−1^ for *E. coli* and *S. aureus*, respectively (Fig. 7c and d). Both the biosynthesized NPs were found to have a significant activity against both the organism, although improved activity was obtained with Ag NPs against *E. coli*.

### 3.4. Study of topological changes in treated bacteria

**Fig. 8:**
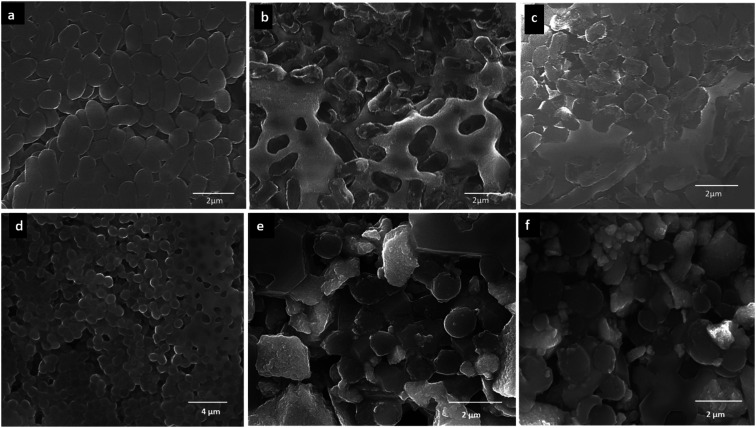
SEM micrographs of treated bacteria at concentration obtained as its MIC (a) *E. coli* control (untreated cells); (b) *E. coli* treated TiO_2_ NPs; (c) *E. coli treated* Ag NPs; (d) *S. aureus* control (untreated cells); (e) *S. aureus* treated TiO_2_ NPs; (f) *S. aureus* treated Ag NPs.

Morphological changes induced by synthesized TiO_2_ and Ag NPs to the test bacteria were further studied by SEM. The control (untreated) *E. coli* cells, appeared as normal rod-shaped with a consistent and smooth cell surface (Fig. 8a). Although, treated *E. coli* with both the NPs was not found intact, with irregularities at cell surfaces seen. The Ag NPs treated cells were seen more damaged than that of the TiO_2_ NPs treated cells. The treatment of *E. coli* cells with TiO_2_ NPs produced a mild alteration (Fig. 8b), whereas Ag NPs treated *E. coli* was severely affected (Fig. 8c). Simultaneously, the untreated *S. aureus* cells (control) were normal in shape *i.e.*, cocci with smooth cell surface (Fig. 8d). The treated *S. aureus* cells on the contrary were seen with irregularities and distorted cell surface. Both the TiO_2_ and Ag NPs had nearly similar outcome on the Gram-positive bacteria (Fig. 8e and f).

### 3.5. Antiproliferative activity

**Fig. 9:**
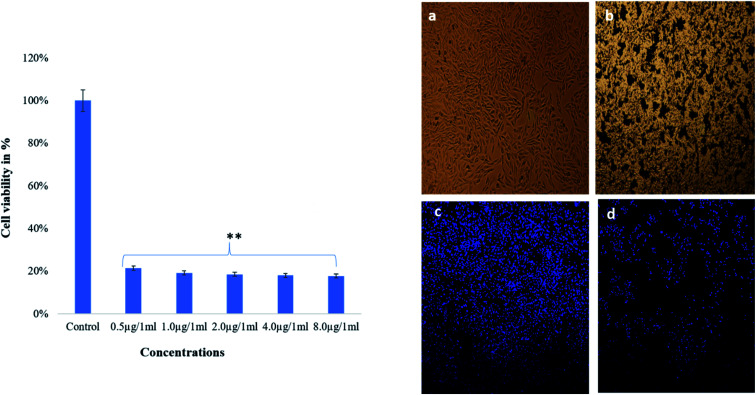
Cell viability of HCT-116 cells by MTT assay on treatment with TiO_2_ NPs after 48 h and cell morphology analysis (a) control; & (b) treated with 8.0 μg mL^−1^, analyzed by light microscope. (c) Control, & (d) treated 8.0 μg mL^−1^ analyzed by confocal scanning microscope. Data are the mean ± SD of three different experiments. Difference between two treatment groups were analysed by Student's *t* test where **p < 0.01, *p*-values were calculated by Student's *t*-test. No changes were observed in control group.

The dose-depended effects on cancer cells survivability was found as examined by MTT assay. The treatment of TiO_2_ NPs showed strong cytotoxic effects on cancer cell viability as more majority of the cells were found dead after treatments of lower 0.5 μg mL^−1^ (Fig. 9). TiO_2_ NP treated cells showed significant alteration in structure and the cell nucleus as revealed by light and confocal microscopy (Fig. 9b and d). It was clearly indicated that nucleus has disintegrated, and also nuclear condensation was observed, along with the death of many cancer cells. It was observed that TiO_2_ NPs-treatment caused significant loss of nuclear staining in the HCT-116 cells as seen by DAPI staining as compared to control cells (Fig. 9a and c).

**Fig. 10:**
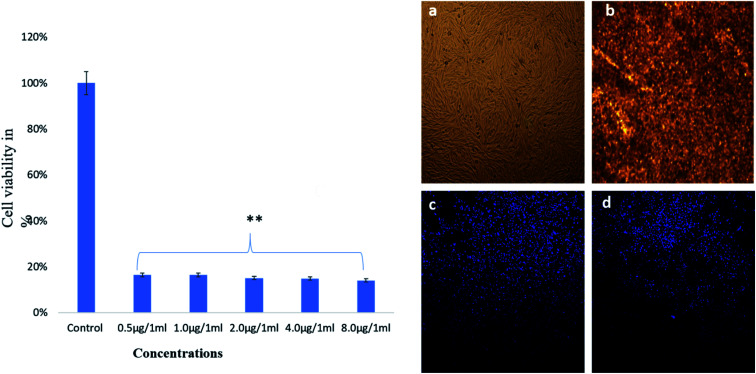
Cell viability of HCT-116 cells by MTT assay on treatment with Ag NPs after 48 h and cell morphology analysis (a) control; & (b) treated with 8.0 μg mL^−1^, analyzed by light microscope. (c) Control, & (d) treated 8.0 μg mL^−1^ analyzed by confocal scanning microscope. Data are the mean ± SD of three different experiments. Difference between two treatment groups were analysed by Student's *t* test where **p < 0.01, *p*-values were calculated by Student's *t*-test. No changes were observed in control group.

The treatment of AgNPs also showed strong cytotoxic effects on cancer cells viability as more majority of the cells were found dead after the treatment of 0.5 μg mL^−1^ (Fig. 10). AgNPs exhibited significant deformities in cell morphology and the nucleus as revealed by light and confocal microscopy (Fig. 10b and d). The clear evidence of disintegration and condensation of nucleus was observed and as many cells were found dead. The Ag NPs-treatment caused significant loss of nuclear staining in the HCT-116 cells as seen by DAPI staining (Fig. 10d) with compared to control cells (Fig. 10a and c).

## 4. Discussion

The phenotypic analysis of mushroom collected from the Kashmir Himalayas indicated to be a brown-rot fungus namely *F. pinicola*, which fills Basidiomycota, Agaricomycetes, Fomitopsidaceae. This common fungal species is a wood colonizing saprotroph found in boreal and temperate forests of the Northern Hemisphere (Fig. 1a). It has a heterothallic sexual system having two haploid mycelia which produce a dikaryotic mycelium to complete the sexual cycle.^22^

Edible mushrooms are known to harbor various bioactive compounds with enormous activities like antioxidant, anti-inflammatory, antimicrobial, hepato protective, hypotensive and anticancer activities.^23^ Based on these properties, an attempt was made to synthesize TiO_2_ and Ag NPs by using the cell free extract of *F. pinicola* as a reducing agent. The production of TiO_2_ and Ag NPs was indicated by examining the color change in the reaction mixture which was confirmed by the XRD spectra. The peak intensity of both TiO_2_, and Ag NPs was studied and observed as per the previous studies by Rajakumar *et al.* and Sriramulu and Sumathi, respectively.^21,24^ The FT-IR spectra showed several functional groups presence in mushroom species assisting TiO_2_ and Ag NPs formation.^25,26^ The study showed the presence of various components related to amino, methyl and hydroxyl group present in mushroom that assist in transformation of titanium oxide and silver nanoparticles. The samples using DRS-UV showed presence of isolated Ti^4+^ species corresponding to tetrahedral coordination^27^ and Ag NPs, exhibited presence of three kinds of Ag species indicating the variation in coordination environments.^28^ SEM and TEM morphology of TiO_2_ and Ag NPs showed irregular shape and size^29^ and agglomeration of nanoparticles with distribution of small spherical nanoparticles (10–30 nm),^30^ respectively.

Broth dilution method was used to evaluate the toxicity of a synthesized TiO_2_ and Ag NPs to bacterial cells and hence the susceptibility of the cells in this mixture. The antimicrobial action of Ag NPs has been well studied and successfully used in the pharmaceutical industries for healing of wounds and as antibiotic applications.^31^ TiO_2_ NPs has also been reported for significant reduction in the number of *Pseudomonas* colonies.^32^ The current study is also supported by the studies carried out by Swathi *et al.* presenting the green synthesized TiO_2_ NPs, against Gram-negative and positive organisms, resulting in enhanced activity against Gram negative bacteria.^33^

This study was aimed to find the antibacterial potential of biologically synthesized TiO_2_ and Ag NPs against *E. coli* and *S. aureus.* Both the biosynthesized NPs were found to have a significant activity against both the organism, although improved activity was obtained with Ag NPs against *E. coli*. The obtained results are in agreement with the number of studies conducted previously stating the antibacterial activity of biosynthesized Ag NPs.^7–11,31^

Morphological changes induced by synthesized TiO_2_ and Ag NPs to the test bacteria indicated that the Gram negative *E. coli* cells were more adversely affected as that of the Gram positive *S. aureus*. The achieved results demonstrated that the greater activity of Ag NPs, synthesized by green approach could be due to their spherical shape and smaller size of 10–30 nm, as compared to TiO_2_ NPs having a larger size of 80–120 nm. The elevated activity might also be facilitated by the efficient attachment of Ag NPs, to the bacterial cell surface, and hence play a crucial role in obtaining significant antibacterial activity. However, many studies have been carried out for the antimicrobial action of Ag NPs, still the exact mechanism of action is not clear.^34,35^ Various studies hypothesized the interaction of electrostatic forces between the negatively charged bacterial cell surface and the positively charged NPs.^36–38^ Additionally, the antimicrobial efficiency of NPs can be also owed to many hindered potential metabolic reactions, like inactivated proteins and enzymes, DNA degradation and so on.^11,39^ Ag NPs have been recorded to interact with the proteins having phosphorous and sulfur moieties as the cell component and genetic materials.^40–42^ Therefore, the successful application of these NPs depends on the controlled size and shape, which offers the enhanced surface area, having a huge impact on various cellular and metabolic processes.^36^

The impact of TiO_2_ and Ag NPs was studied both morphologically using light and confocal microscope and quantitatively by MTT assay. The dose-depended effects on cancer cells survivability was found as examined by MTT assay. The clear evidence of disintegration and condensation of nucleus was observed and as many cells were found dead. The Ag NPs-treatment caused significant loss of nuclear staining in the HCT-116 cells as seen by DAPI staining. The treatment of nanoparticles also showed strong cytotoxic effects on cancer cells viability as more majority of the cells were found dead after treatments of nanoparticles. The mechanism through which cancer cells die could be attributed to program cell death, as we have seen that cancer cells were undergone nuclear disintegration, and DNA fragmentation after nanoparticles treatment. This programmed cell death or apoptosis is major marker for the cancer cell death. However, it is of paramount importance to study the molecular pathways involved in NPs mediated cancer cell death. Interestingly, there are several reports of NPs, which are known to induce fragmentation and disintegration of nucleus in cancer cells.^43–45^

## 5. Conclusion

Therefore, present study of green synthesis of NPs offers convincing antibacterial activity against the Gram-negative and Gram-positive bacteria, which could be a boon for treating the infectious diseases and can be utilized in food industry to minimize the contamination and extend the duration for preservation. Additionally, the cytotoxic activity holds the potential as a promoter for human colon cancer therapy.

## Author contributions

S. Rehman, conceptualized and designed the study, Suriya Rehman, Romana Farooq, Sarah Mousa Asiri, Firdos Alam Khan, Rabindran Jermy, M. A. Ansari and Zainab Alsalem carried out the experiments and prepared all figures. Suriya Rehman, Vijaya Ravinayagam, M. A. Shah and Reem Al Jindan discussed the results and wrote the first draft. Firdos Alam Khan, Suriya Rehman and Rabindran Jermy revised and edited the manuscript. All the authors have read and approved the final manuscript.

## Finance support

Financial support is acknowledged from Deanship of Scientific Research, Imam Abdulrahman Bin Faisal University, Dammam, Saudi Arabia, under Project No. 2019-072-IRMC.

## Availability of data and material

The data analyzed are available from the corresponding author upon a request.

## Conflicts of interest

No potential conflict of interest is reported by authors.

## Supplementary Material

RA-010-D0RA02637A-s001
