# Supplementary material for: Using Fomitopsis pinicola for bioinspired synthesis of titanium dioxide and silver nanoparticles, targeting biomedical applications
Source: RSC Adv. 2020 Aug 28;10(53):32137–47. doi: 10.1039/d0ra02637a (PMC9056542; doi:10.1039/d0ra02637a)
Supplement: RA-010-D0RA02637A-s001 [file RA-010-D0RA02637A-s001.pdf]

Electronic Supplementary Material (ESI) for RSC Advances.
This journal is © The Royal Society of Chemistry 2020

Operating *Fomitopsis* pinicola for Bioinspired Synthesis of Titanium Dioxide and Silver Nanoparticles, Targeting Biomedical applications

Dr. Suriya Rehman[1*], Dr. Rabindran Jermy[2*], Ms. Sarah Mousa Asiri[4], Dr. Manzoor A. Shah[3], Ms. Romana Farooq[2], Dr. Vijaya Ravinayagam[5], Dr. Mohammad Azam Ansari[1], Ms. Zainab Alsalem[1], Reem Al Jindan [6], Prof. Firdos Alam Khan[7]

SAMPLING:

Extensive field surveys were conducted in the coniferous forests of Budgam, Ganderbal, Anantnag, Baramullah and Pulwama district of Kashmir valley during 2016-2018 growing seasons of mushrooms.

SITE NAME	ALTITUDE MASL	LATITUDE	LONGITUDE	SITE CHARACTERISTICS	DISTRICT
Mammer	2400 m	34°14′ N	75°01′ E	Open forest areas with coniferous trees	Ganderbal
Kellar	1630 m	33 °46′N	74°46′ E	Open coniferous forests	Pulwama
Gulmarg	2703m	34°03′ N	74°23′ E	Dense forest area with mixed plantation	Baramulla
Yusmarg	2400m	33°50′ N	74°38′ E	Open forests some grassy fields	Budgam
Pahalgam	2740 m	34°01 N	74°31′ E	Dense Coniferous forests	Anantnag
